# Not all antibodies are created equal: total IgG glycosylation and severity of antibody-mediated rejection in kidney transplantation

**DOI:** 10.3389/ti.2026.16461

**Published:** 2026-06-12

**Authors:** Johan Noble, Leandre M. Glendenning, Celine Dard, Anne Bourdin, Marta Crespo, Umberto Maggiore, Ari R. Inwood, Grace C. Carlson, Brian A. Cobb, Paolo Cravedi

**Affiliations:** 1 Translational Transplant Research Center (TTRC), Icahn School of Medicine at Mount Sinai, Precision Immunology Institute, New York, NY, United States; 2 Nephrology, Hemodialysis, Apheresis and Kidney Transplantation Department, University Hospital Grenoble, Grenoble, France; 3 Inserm U 1209, CNRS UMR 5309, Team Cell Dynamics, Immunity, Metabolism & Cancer, Institute for Advanced Biosciences, University Grenoble Alpes Grenoble, France; 4 Department of Pathology, Case Western Reserve University School of Medicine, Cleveland, OH, United States; 5 Etablissement Français du Sang, Grenoble-Alpes, France; 6 Department of Nephrology, Hospital Ddel Mar, Nephropathies Research Group, Hospital del Mar Research Institute, Barcelona, Spain; 7 Nephrology Unit, Department of Medicine and Surgery, University Hospital of Parma, Parma, Italy

**Keywords:** antibody-mediated rejection, glycosylation, immunoglobulin G, N-acetylglucosamine, renal transplant

## Abstract

Antibody-mediated rejection (AMR) is a leading cause of kidney transplant failure and is primarily driven by donor-specific anti-HLA antibodies (DSA), although DSA presence alone does not fully explain the heterogeneity of AMR severity. We prospectively studied 79 kidney transplant recipients from 2 European centers, including 29 with active AMR (aAMR), 29 with chronic-active AMR (caAMR), and 21 controls without rejection, to investigate the association between total Immunoglobulin-G (IgG) glycosylation profiles and AMR. IgG glycosylation was quantified using lectin-based ELISA, assessing relative levels of mannose, core fucose, α2,6-linked sialic acid, and bisecting N-acetylglucosamine (GlcNAc). Bisecting GlcNAc levels were higher in caAMR compared with controls and aAMR (both p < 0.001), while core fucosylation and mannose levels were increased in aAMR and caAMR relative to controls. Higher levels of core fucose, mannose, and α2,6-sialylation were associated with increasing glomerulitis severity. We found that bisecting GlcNAc (PHA-E/Fc) was significantly associated with both higher g- and cg-score (in ordinal models, OR = 2.1 [95% CI: 1.2–4.1; p = 0.017], and OR = 2.0 [95% CI: 1.1–4.3; p = 0.046], respectively). Mannose level (ConA/Fc) was significantly associated with higher g-score (2.1 [1.1–4.2], p = 0.017]). These findings indicate that distinct total IgG glycosylation features are independently associated with specific histological patterns of AMR severity.

## Graphical Abstract

**Figure:**
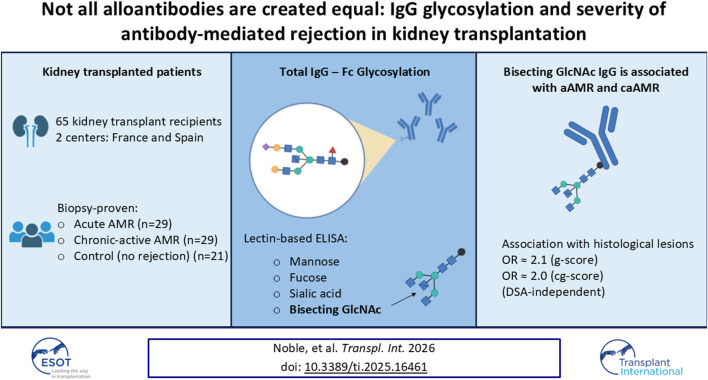

## Introduction

Antibody-mediated rejection (AMR) is a major cause of kidney transplant loss [1]. Although the pathophysiology of AMR is not fully understood, donor-specific anti-HLA antibodies (DSA) are thought to play an important pathogenic role. When present, these antibodies target endothelial graft cells, leading to their direct activation [2], the initiation of the complement cascade, and the recruitment of immune cells [3, 4]. Antibodies can also promote endothelial injury through antibody-dependent cellular cytotoxicity (ADCC) and the release of pro-inflammatory cytokines, both events that are primarily mediated by natural killer (NK) cells [5–8].

While DSA are central to AMR pathogenesis - triggering complement-dependent cytotoxicity (CDC), antibody-dependent cellular cytotoxicity (ADCC), and proinflammatory injury - not all patients with DSA experience graft loss [9]. This disconnect suggests that titer of DSA alone fails to explain clinical outcomes.

Evidence has shown that post-translational modification of antibodies affects their function [10, 11]. The type and level of antibody glycosylation can influence its ability to activate complement and induce ADCC, as well as their stability and circulating half-life [12]. Glycosylation of Immunoglobulin G (IgG) involves the addition of carbohydrate at a conserved site in the Fc region (Asn297), and, less frequently, in the Fab region [13]. Unlike the conserved site in the Fc region, glycan sites within the Fab region are primarily introduced during somatic hypermutation. N-linked glycosylation starts with the transfer of a high mannose glycan from a dolichol-glycan donor in the endoplasmic reticulum (ER) and is critical for protein folding quality control (Supplementary Figure 1A). Upon exit from the ER and entry into the Golgi apparatus, the high mannose structures are matured by sequential removal of all but three mannoses, leaving a pentasaccharide core (Supplementary Figure 1B), and then rebuilt through the addition of N-acetylglucosamine (GlcNAc) residues (Supplementary Figure 1C) and varying degrees of additional capping glycans (Supplementary Figure 1D). This results in complex N-glycans carrying varied amounts of GlcNAc branching, core fucosylation, galactose and sialic acid residues. Glycan structures found on IgG are typically bi-antennary, and may contain bisecting GlcNAc (Supplementary Figure 1D), a branching modification that influences IgG function and enhances Fc receptor binding. Functionally, sialylation (addition of terminal N-acetylneuraminic acid with an α2,6 linkage to the underlying galactose) and core fucosylation (fucose attached with an α1,6 linkage to the asparagine-proximal GlcNAc) are associated with reduced ADCC properties by reducing Fc gamma receptors (FcγR)III affinity [14].

IgG glycosylation profile has been associated with changes in the severity of various autoimmune diseases [15]. A study in kidney transplant recipients showed that low sialylation and high bisecting GlcNAc of anti-donor specific HLA antibodies (DSA) were associated with higher risk of AMR [16]. However, this study assessed only post-translational modifications in IgG3. Herein, we hypothesized that total IgG glycosylation profile may be associated with the risk and the severity of both AMR and caAMR.

## Materials and methods

### Study population

We used serum samples from prospectively followed-up kidney transplant recipients from two centers (Grenoble-Alpes, Grenoble, France and Hospital Del Mar of Barcelona, Barcelona, Spain) between January 2016 and May 2024 with a biopsy-proven AMR and control kidney transplant patients with no signs of microvascular inflammation (MVI). All samples were collected at the time of biopsy. All control biopsies were per protocol, taken at 3 months, 1, and 3 years post-transplant. All biopsies were scored according to Banff 2022 and the indications were protocol biopsies or for cause [17]. In brief, AMR was defined by the presence of MVI, i.e., glomerulitis (g) and peritubular capillaritis (ptc). If the MVI score was 2 or above, AMR was confirmed by the presence of C4d deposition in peritubular capillaries and vasa recta and/or by the presence of HLA DSA. If other features of AMR, such as acute thrombotic microangiopathy were present, but the MVI score was below 2, AMR was confirmed by the presence of C4d deposition, regardless of DSA status. C4d deposition was quantified using anti-C4d antibody on formalin-fixed, frozen paraffin-embedded sections. Chronic active AMR (caAMR) was defined by the presence of active lesions (including C4d) and chronic lesions (transplant glomerulopathy (cg) and/or peritubular capillary multilayering).

All patients signed an informed consent form. For Grenoble Hospital, all medical data were collected from the Grain-DB database [CNIL (French National committee for data protection) approval number 1987785v0 for the Nephrology and Kidney transplantation department of Grenoble-France]. The study was also approved by the Institutional Review Board of the Consorci Mar Parc de Salut de Barcelona (Research Project No. 2020/9117/I) and was conducted in accordance with the principles of the Declaration of Helsinki and the Declaration of Istanbul.

### Anti-HLA antibody measurement

Anti-HLA antibodies were measured at the time of biopsy in all patients using One Lambda-Thermofisher Labscreen, Class I, and Class II or Lifecodes LSA Class I and/or Class II test (Immucor GTI Diagnostics, Inc®). Antibody specificity was identified using a Luminex® 200 Instrument (Luminex Corporation, Austin, TX). The MFI threshold of positivity for a DSA was 500 for patients followed-up in Grenoble and 750 for those Barcelona. The sera used for the post-translational analysis were leftovers from the anti-HLA analysis at the Etablissement Français du Sang (EFS) of Grenoble, HLA department and study-dedicated sera for patients enrolled in Barcelona.

### Immunoglobulin G purification

We purified total serum IgG using Protein A IgG Purification Kit (Pierce™, Thermo Scientific™, CAT: 44667) and following manufacturer’s instructions. Briefly, the procedure consisted of passing the sera sample into the column and then eluting the IgG bound to the resin using an elution buffer (pH 2.8). For each sample, IgG concentration was measured by absorbance at 280 nm and purity was confirmed using SDS-PAGE.

### Quantification of glycosylation by lectin ELISA

Purified IgG were diluted to 1 ng/μL in PBS, plated into a 96-well high-binding ELISA plate (Microlon High Binding; Greiner BioOne) and incubated overnight at 4 °C. The plate was blocked with carbohydrate free blocking solution (Vector Labs) for 1 h at room temperature. Biotinylated lectins (Vector Labs) were diluted to 1 μg/mL in carbohydrate-free blocking solution and incubated for 1 h at room temperature. Different biotinylated lectins were used: Sambucus nigra agglutinin (SNA): binds to α2,6-linked Neu5Ac and is used to quantify the sialylation of IgG. Concanavalin A (ConA): binds to high mannose and hybrid N-glycans and correlates with more immature IgG. Lens culinaris agglutinin (LCA): binds to core fucosylated N-glycans. Phaseolus vulgaris Erythroagglutinin (PHA-E) binds to bisecting GlcNAc to quantify bisecting GlcNAc on IgG. Anti-Fc measures the amount of immobilized IgG to normalize the results. The amount of IgG coated was 0, 100ng and 200 ng to assess saturation of the signal, and we thereafter used the results of 100 ng for consistency. Signal was detected using europium-conjugated streptavidin (Perkin Elmer) and time-resolved fluorescence as measured in a Victor Nivo plate reader.

### Statistical analysis

Normally distributed quantitative variables were expressed as mean ± standard deviation (SD). Non-normally distributed quantitative variables were expressed as median [interquartile ranges (IQR)]. All glycosylation values were non-normally distributed (p-value from Shapiro-Wilk Test <0.005). Qualitative variables were expressed as numbers and percentages. For statistical comparison between multiple groups, we used Kruskal-Wallis test. For statistical comparisons between two groups, we used unpaired Mann-Whitney U Tests. The chi-squared test was used to compare categorical data. Statistical significance was defined as a two-sided p-value <0.05.

To identify factors associated with each ordinal outcome variable (i.e., scores of Banff components), we employed ordinal logistic regressions using the proportional odds model using the *clm* function from the ordinal R package (R version 4.4.2, https://www.r-project.org). Ordinal logistic regression models for g- cg- and pc- scores were fitted for each glycan, and adjusted for DSA, recipient age, sex, delay of biopsy since transplantation, and eGFR at biopsy whenever they were statistically significant to avoid overfitting given the limited sample size. These covariates were included, as they may affect both glycosylation and histological outcomes independently of any direct glycosylation-mediated effect [18, 19]. To visualize the ordinal regression model for cg score, we plotted the fitted predicted probability for each cg score level as a function of standard unit change of bisecting GlcNAc (PHA-E/Fc).

## Results

### Baseline characteristics of the study population

The study included 79 kidney transplant recipients, 29 (36.7%) with biopsy-proven aAMR, 29 (36.7%) with caAMR, and 21 (26.6%) controls with no histological signs of rejection at per-protocol biopsy (Table 1). There was no difference among patients with aAMR, caAMR and controls in the main characteristics such as the age at the time of biopsy, gender, previous transplantation number, type of donor, HLA mismatches, history of diabetes, and induction therapy. The median time between the transplantation and the biopsy was 0.9 years [0.1–4.8] in the aAMR group, 3.9 [2.9–12.3] for the caAMR group, and 3.2 [0.3–3.6] for the control group, p = 0.004. The immunosuppressive regimen consisted of tacrolimus, mycophenolate mofetil, and steroids for all patients. Patients with aAMR or caAMR had higher proteinuria than controls (Table 1).

**TABLE 1:** Baseline characteristics of study participants.

Characteristics	CTL (N = 21)	aAMR (N = 29)	caAMR (N = 29)	*p-value*
Age - years	51.7 ± 12	43.7 ± 17	43.4 ± 17	0.179
Gender M/F ratio	3.2	0.9	1.7	0.125
History of previous transplantation – N (%)	0	8 (27.6)	3 (10.3)	0.065
Nephropathy – N (%) Diabetes Drug-related Nephrotic disease Genetic Uropathy Unknown Glomerulonephritis TMA Hypertensive nephropathy Interstitial nephropathy Acute tubular necrosis Polycystic disease	1 (4.7) 1 (4.7) 1 (4.7) 1 (4.7) 1 (4.7) 7 (33.2) 1 (4.7) 1 (4.7) 2 (9.5) 2 (9.5) 1 (4.7) 4 (19)	0 0 3 (10.3) 1 (3.4) 2 (6.9) 7 (24.1) 7 (24.1) 1 (3.4) 3 (10.3) 1 (3.4) 0 2 (6.9)	2 (6.9) 0 2 (6.9) 0 3 (10.3) 5 (17.24) 6 (20.7) 1 (3.4) 3 (10.3) 2 (6.9) 0 4 (13.8)	0.522
Time of biopsy - months after transplant	38.5 [3–43]	10.9 [2–58]	47.3 [35–149]	0.004
PRA (%)	11 ± 28	49 ± 44	30 ± 45	0.021
Living donor – N (%)	4 (19)	7 (24.1)	6 (6.9)	0.902
Donor age - years	55.2 ± 14	49.5 ± 16	45.8 ± 16	0.192
HLA mismatch A/B/DQ - number	5.0 [3–6]	6.0 [5–7]	5.5 [5–7]	0.218
History of diabetes – N (%)	1 (4.7)	0	2 (6.9)	0.329
Anti-thymoglobulin induction – N (%)	9 (42.7)	21 (72.4)	20 (68.9)	0.725
MVI ≥2 – N (%)	0	24 (82.7)	28 (96.5)	<0.001
C4d positivity– N (%)	0	22 (75.8)	10 (34.5)	<0.001
Donor-specific antibodies – N (%)	1 (4.7)	23 (79.3)	21 (72.4)	<0.001
cg-score positivity– N (%)	0	0	29 (100)	<0.001
eGFR at the time of biopsy – mg/dL	43 [31–63]	45 [32–66]	36 [23–49]	0.145
Proteinuria at rejection – g/g	0.1 [0.1–0.4]	0.3 [0.1–0.7]	0.8 [0.3–2.0]	<0.001

FSGS, Focal segmental glomerulosclerosis; HLA, Human Leukocyte Antigen; IgAN, IgA nephropathy; MVI, microvascular inflammation; PRA, panel reactivity antibody; eGFR, estimated glomerular filtration rate; TMA, thrombotic microangiopathy.

Twenty-three aAMR patients (79.3%) had donor-specific antibody (DSA) at the time of the biopsy, compared to 21 (72.4%) in the caAMR group (p = 0.539). One patient in the control group had DSA. The number of DSA was also similar between the two rejection groups (1.5 ± 0.9 vs. 1.4 ± 0.7, p = 0.539) and most of DSA were class II (81% versus 85%, p = 0.731) for aAMR and caAMR, respectively. The mean fluorescence intensity (MFI) of the immunodominant DSA was also similar (8226 ± 6549 vs. 4915 ± 5968, p = 0.192).

### Associations between IgG glycosylation and the risk of AMR

**FIGURE 1:**
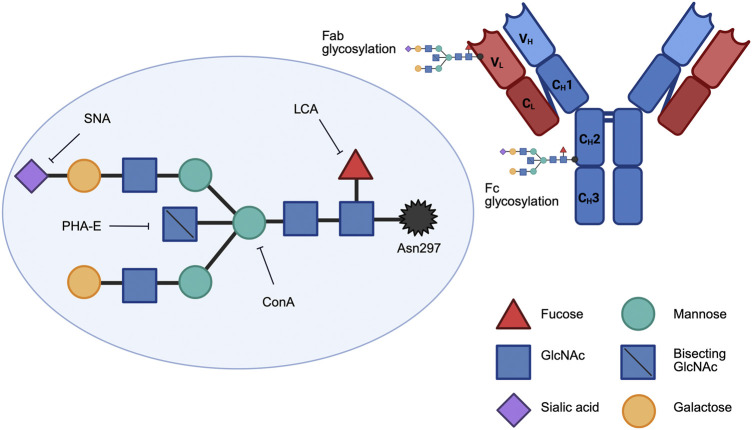
Representation of Immunoglobulin G glycosylation sites and detection. The antibody regions are labeled as C_H_1, C_H_2, C_H_3 (constant heavy domains), V_H_ (variable heavy), V_L_ (variable light), and C_L_ (constant light). A critical glycosylation site at *Asn297* in the Fc region is shown, along with Fab glycosylation. The figure also illustrates key glycan components, including fucose, sialic acid, mannose, bisecting GlcNAc, and galactose. Additionally, lectins with binding specificity (LCA, PHA-E, SNA, ConA) are indicated, suggesting their utility in glycan analysis.

The quantification of IgG glycan composition (relative amounts of mannose, fucose, sialic acid, and bisecting GlcNAc) was assessed using the ConA, LCA, SNA, and PHA-E lectins, respectively, and normalized with the Fc levels (Figure 1). We found a positive correlation between mannose, core fucose, α2,6-linked sialic acids. In contrast, bisecting GlcNAc was not significantly associated with the other glycoforms (Supplementary Figure 2). There was no correlation between the glycan level and the total amount of IgG (data not shown).

**FIGURE 2:**
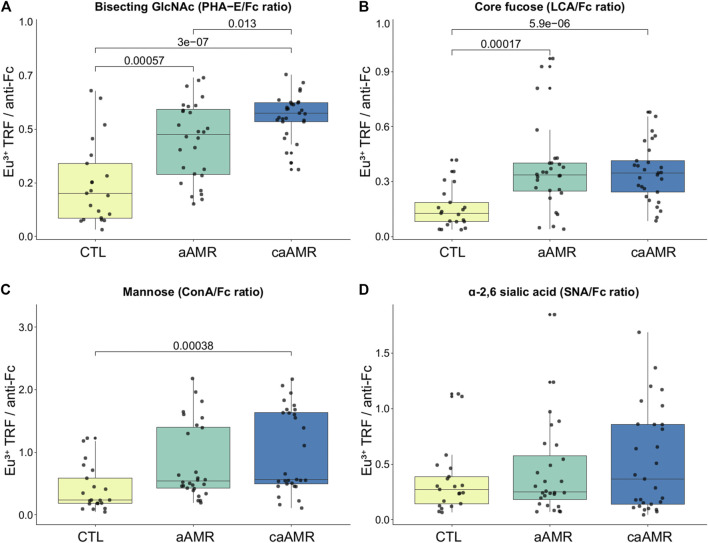
Associations between IgG post-translational modifications and the presence of antibody-mediated rejection. Box plots comparing the amounts of bisecting GlcNAc (PHA-E/Fc ratio in panel **(A)**, core fucose (LCA/Fc ratio in panel **(B)**, mannose (ConA/Fc ratio in panel **(C)**, and α2,6-sialylation (SNA/Fc in panel **(D)** among control patients, patients with acute antibody-mediated rejection and chronic-active antibody mediated rejection. p-values <0.05 is indicated for each comparison. aAMR: acute Antibody-Mediated Rejection; caAMR, chronic active Antibody-Mediated Rejection; CTL, control patients, ConA, Concanavalin A; Eu^3+^ TRF/anti-Fc, europium-conjugated time-resolved fluorescence normalized to IgG; LCA, Lens culinaris agglutinin; SNA, Sambucus nigra agglutinin; PHA-E, Phaseolus vulgaris Erythroagglutinin.

We first compared the different IgG glycoforms among the 3 groups. The amounts of bisecting GlcNAc (PHA-E/Fc), core fucose (LCA/Fc), and mannose (ConA/Fc) were significantly lower in the control group than in the caAMR group. Bisecting GlcNAc (PHA-E/Fc) was significantly higher in the caAMR and aAMR groups compared to controls, and significantly higher in caAMR compared to aAMR patients (Figure 2A). Core fucose (LCA/Fc) was significantly higher in both caAMR and aAMR groups compared to controls (Figure 2B). Mannose (ConA/Fc) was significantly higher in caAMR than in controls (Figure 2C). There was no statistical difference among the 3 groups in the α2,6-sialylation (SNA/Fc) levels (Figure 2D). When gathering aAMR and caAMR, we found higher level of bisecting GlcNAc (p < 0.001), of fucosylation (p < 0.001), and of Mannose (p < 0.001) in rejection patients compared to controls while sialylation levels were similar (p = 0.41).

### Associations between IgG glycosylation and the severity of rejection

**FIGURE 3:**
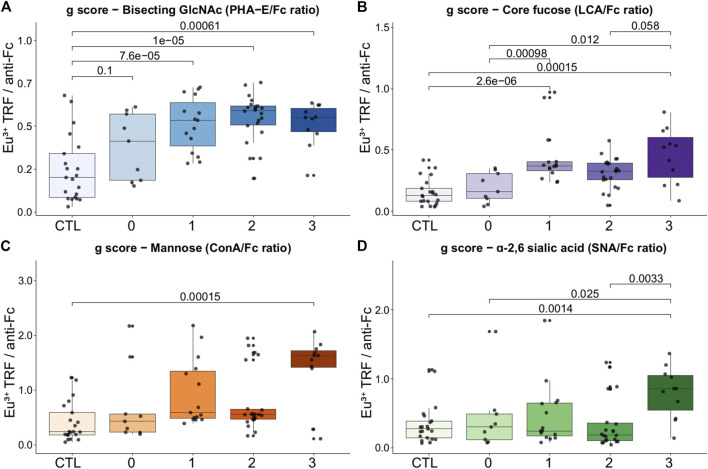
Associations between IgG post-translational modifications and the severity of rejection. Box plots comparing the levels of bisecting GlcNAc (PHA-E/Fc ratio in panel **(A)**, core fucose (LCA/Fc ratio in panel **(B)**, mannose (ConA/Fc ratio in panel **(C)**, and α2,6-sialylation (SNA/Fc in panel **(D)** with the severity score of glomerulitis (g-score). p-values <0.05 is indicated for each comparison. ConA, Concanavalin A; cg, chronic glomerulopathy; c4d, complement factor 4d; g, glomerulitis; Eu^3+^ TRF/anti-Fc, europium-conjugated time-resolved fluorescence normalized to IgG; LCA, Lens culinaris agglutinin; PHA-E, Phaseolus vulgaris Erythroagglutinin; ptc: peritubular capilaritis; SNA, Sambucus nigra agglutinin.

We then compared the amount of each glycoform with the severity of AMR using each of the AMR parameters listed in the Banff score (0–3), i.e., g-, cg-, and ptc- scores and C4d deposition intensity (0–3). We used cg-score to quantify the chronicity of lesions. We found no association between bisecting GlcNAc (PHA-E/Fc) and g-score increasing severity (Figure 3A). Core fucose (LCA/Fc), mannose (ConA/Fc) and α2,6-sialic acids (SNA/Fc) levels in IgG were increased in samples from patients with the highest g-scores compared to those with lower scores (Figures 3B–D). Within AMR cases, core fucosylation was increasing with the severity of glomerulitis (Figure 3B). We also found that patients with highest cg-score had increased bisecting GlcNAc (PHA-E/Fc), core fucose (LCA/Fc), mannose (ConA/Fc), and sialylation (SNA/Fc; Supplementary Figure 3) levels. Finally, there was a correlation between ptc-score severity and IgG glycan levels (Supplementary Figure 4).

### Association between IgG glycosylation and DSA

Not all patients in our cohorts had DSA. Therefore, we tested the association between IgG glycosylation and DSA. Among the different glycosylation tested, we found no significant correlation between total IgG glycosylation and the presence of DSA in patients with AMR (Supplementary Figure 5A–D). In particular, the level of bisecting GlcNAc was not different between aAMR and caAMR patients with or without DSA (Supplementary Figure 5A).

**FIGURE 4:**
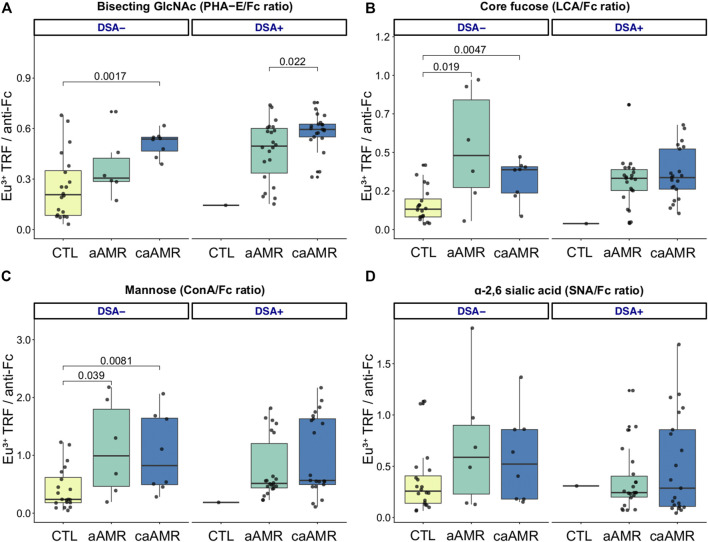
Associations between IgG post-translational modifications and the presence of antibody-mediated rejection according to the presence or absence of Donor-specific antibodies (DSA). Box plots comparing the amounts of bisecting GlcNAc (PHA-E/Fc ratio in panel **(A)**, core fucose (LCA/Fc ratio in panel **(B)**, mannose (ConA/Fc ratio in panel **(C)**, and α2,6-sialylation (SNA/Fc in panel **(D)** among control patients, patients with acute antibody-mediated rejection and chronic-active antibody mediated rejection, according to the presence or absence of circulating DSA at the time of the biopsy. aAMR, acute Antibody-Mediated Rejection; caAMR, chronic active Antibody-Mediated Rejection; CTL, control patients, ConA, Concanavalin A; DSA, Donor specific antibodies; Eu^3+^ TRF/anti-Fc, europium-conjugated time-resolved fluorescence normalized to IgG; LCA, Lens culinaris agglutinin; SNA, Sambucus nigra agglutinin; PHA-E, Phaseolus vulgaris Erythroagglutinin.

We repeated the analyses comparing glycoform levels in controls, aAMR, and caAMR cohorts, by stratifying patients based on the presence of DSA. In DSA negative patients, caAMR remained significantly higher than controls for bisecting GlcNAc, total mannose and core fucose while aAMR remained significantly associated with total mannose and core fucose (Figures 4A–C). In DSA positive patients, bisecting GlcNAc was higher in caAMR compared to aAMR patients (Figure 4A). α2,6-sialylation was not significantly different between controls and AMR conditions, regardless of DSA status (Figure 4D).

### Multivariable analysis of factors associated with histologic features of AMR

Finally, we tested whether the IgG glycosylation and AMR severity are independently associated.

**FIGURE 5:**
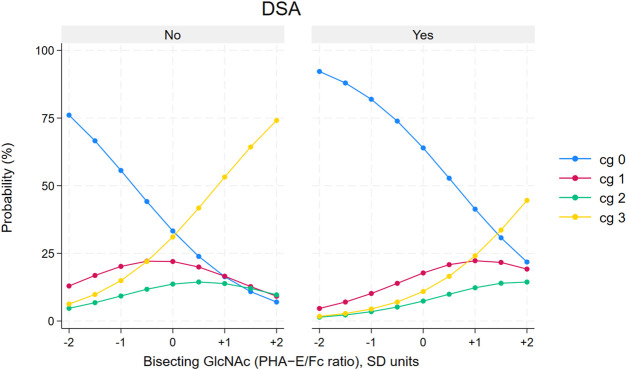
Visual representation of the multivariable ordinal logistic regression for chronic glomerulopathy severity (cg score), including both DSA and bisecting GlcNAc. Predicted probabilities of each cg-score (0–3) derived from the fitted multivariable ordinal logistic regression including bisection GlcNAc (PHA-E/Fc ratio) and DSA. The x-axis represents standardized bisecting GlcNAc levels. The left panel shows DSA-negative patients; the right panel shows DSA-positive patients. Lines represent predicted probabilities of cg = 0 (blue), cg = 1 (red), cg = 2 (green), and cg = 3 (yellow). In DSA negative patients (left panel), high bisecting GlcNAc values (+2 SD units) are associated with a large difference between the probability of cg = 3 and cg = 0; this difference vanishes in DSA positive patients (right panel).

We found that bisecting GlcNAc (PHA-E/Fc) was significantly associated with both higher g- and gc-scores (in ordinal models, OR = 2.1 [95% CI: 1.2–4.1; p = 0.017], and OR = 2.0 [95% CI: 1.1–4.3; p = 0.046], respectively). Mannose level (ConA/Fc) was significantly associated with higher g-score (2.1 [1.1–4.2], p = 0.017] (Table 2). Core fucosylation and sialylation were not significantly associated with AMR severity in multivariable analyses. In Table 2 DSA might seem protective with respect to cg-score (OR less than 1), but this multivariable finding is artifactual as shown by the visual inspection of the predicted probability from the ordinal regression model (Figure 5). In DSA-negative patients, high bisecting GlcNAc predicted severe glomerulopathy (cg = 3), while low levels were protective (cg = 0). This dose-response relationship was attenuated in DSA-positive patients, where high bisecting GlcNAc barely influenced glomerulopathy severity. The apparent protective effect of DSA in multivariable models is therefore artifactual, likely reflecting DSA’s dominant role that masks glycosylation-mediated injury. Bisecting GlcNAc and DSA appear to represent independent but competing pathogenic mechanisms.

**TABLE 2:** Multivariable ordinal regression analysis of the association between clinical variable, IgG post-translational modifications, and AMR severity scores in the overall cohort.

​	g-score		ptc-score	cg-score		
Variables	OR (95% CI)	p-value	OR (95% CI)	p-value	OR (95% CI)	p-value
Presence of DSA	​	​	​	​	​	​
Bisecting GlcNAc	2.1 (1.2–4.1)	0.017	​	​	2.0 (1.1–4.3)	0.046
Core fucose	​	​	​	​	​	​
α2,6 sialic acid	​	​	​	​	​	​
Mannose	.1 (1.1–4.2)	0.017	​	​	​	​
Recipient age (years)	​	​	​	​	​	​
Recipient gender (male)	​	​	4.1 (1.2–15.7)	0.027	3.7 (1.1–13.8)^&,£,§^	0.038
eGFR (ml/min/1.73m^2^)*	​	​	​	​	0.9 (0.9–0.9) ^$,&,£^	0.02
Delay post-transplant (year)	​	​	​	​	1.1 (1.0–1.2) ^$,&,£^	0.009
DSA	​	​	​	​	​	​

Bisecting GlcNAc (PHA-E/Fc ratio); Core fucose (LCA/Fc ratio); α2,6 sialic acid (SNA/Fc ratio); mannose (ConA/Fc ratio), DSA: Donor specific Antibody; eGFR: estimated Glomerular Filtration Rate. *At rejection. ^§^Model with bisecting GlcNAc. ^$^Model with Core fucosylation. &Model with Sialylation. ^£^Model with Mannose.

## Discussion

In this study, we found an association between total IgG core-fucosylation, bisecting GlcNAc, and detectable mannose, and the presence of biopsy-proven AMR in kidney transplant recipients. In contrast, IgG α2,6-sialylation was not associated with AMR in our cohort. Moreover, bisecting GlcNAcylation was significantly associated with both glomerulitis (g-score) and chronic glomerulopathy (cg-score). Interestingly, this association appeared independent of DSA status and may represent a competing pathogenic mechanism.

An inverse association between DSA presence and cg-score emerged in the multivariable model. Exploratory interaction analyses suggest that this finding likely reflects effect-modification rather than a protective role of DSA. The association between higher bisecting GlcNAc levels and severe chronic glomerulopathy was mainly observed in DSA-negative patients, whereas in DSA-positive individuals, glycosylation levels did not substantially alter the predicted probability of severe lesions. These findings suggest that the association between IgG glycosylation and chronic glomerulopathy may be primarily observable in the absence of detectable DSA, whereas in DSA-positive patients, the pathogenic signal associated with DSA may dominate and attenuate the relative contribution of glycosylation levels.

Our findings confirm and expand results from prior smaller studies. Pernin et al. performed mass spectrometry on isolated IgG3 *de novo* DSA and showed that higher bisecting GlcNAc glycosylation was higher in AMR patients overall compared to patients DSA+, but without histological AMR [16]. If mass spectrometry allows a precise characterization of glycoforms, it can also exhibit higher technical variability compared to other methods, potentially affecting reproducibility [20]. ELISA, on the contrary, is well-suited for large-scale studies due to its simplicity and ability to process many samples simultaneously, is generally less expensive and requires less specialized equipment compared to mass spectrometry, and can be standardized across different laboratories, enhancing reproducibility [21].

Our findings are in line with the literature about bisecting GlcNAc. Bisecting GlcNAcylation levels can modify the Fc activity of IgG by influencing its binding affinity to FcγRs. Specifically, the presence of a bisecting GlcNAc moiety has been shown to inhibit Fc core-fucosylation and enhance the binding of Fc to FcγRIIIa, an activating Fcγ receptor [22, 23]. This enhancement in binding affinity can lead to increased ADCC activity, which conceivably results in increased glomerular injury induced by HLA and non-HLA antibodies.

In the study by Pernin et al., sialylation of IgG3 DSA was found to be associated with a reduced risk of AMR. Sialylation of total IgG assessed at the time of transplantation was also associated with reduced AMR risk during the follow-up in another study [24]. In our cohort, we did not find any correlation between IgG sialylation and the presence of AMR. Together, these data suggest a reduced activity of HLA and non-HLA antibodies in kidney transplantation when associated with a higher sialylation. This hypothesis is supported by the literature, as sialylation of the IgG Fc domain has been shown to impair complement-dependent cytotoxicity (CDC) by reducing the binding of C1q [25], decreasing ADCC (in the presence of core fucosylation) by reducing the binding affinity of IgG to FcγRIIIa [26], and exhibiting anti-inflammatory effects [27].

The significance of our data extends to individuals with autoimmune diseases, where alterations in IgG glycosylation have been well-documented. For instance, in rheumatoid arthritis, there is a notable decrease in galactosylation and sialylation, and increased fucosylation in IgG during active phases of the disease [28], suggesting that these glycosylation changes can serve as biomarkers for disease activity and therapeutic response. Similarly, systemic lupus erythematosus patients exhibit decreased IgG galactosylation and sialylation, increased bisecting GlcNAc, and decreased core fucose [29, 30]. These glycosylation changes are associated with disease activity and specific clinical manifestations, such as nephritis and the onset of antinuclear antibodies. IgG glycosylation patterns, including sialylation, bisecting GlcNAcylation, fucosylation, and mannosylation, are significantly altered in various other autoimmune and inflammatory diseases, and these changes have been proposed as potential biomarkers for disease activity and severity [31, 32].

One limitation of our study is that we analyzed total IgG glycosylation, instead of DSA-specific IgG glycosylation, as they may theoretically differ. Of note, one single patient in the control group had DSA, further limiting the power of our analyses to infer the impact of total IgG glycosylation, DSA and AMR severity. However, Sonneveld et al. demonstrated that the Fc-glycosylation profiles of total serum IgG1 and IgG3 were highly similar to those of antigen-specific anti-K antibodies [33]. In that study, the authors analyzed the Fc-tail glycosylation of both total serum and antigen-specific IgG1 and IgG3 using mass spectrometry and found that the glycosylation patterns were consistent across both types, with little individual variability. Using an integrated method for simultaneous protein quantitation and glycosylation profiling of antigen-specific IgG1, Gijze et al. also found that the glycosylation profiles of antigen-specific IgG1 are comparable to those of total IgG1 in a large coronavirus vaccination cohort [21]. Of note, we found that the differences in bisecting GlcNAcylation persist in patients with and in those without DSA, suggesting that although DSA may have a unique glycosylation profile, some key immunomodulatory glycoforms are probably shared across antibody specificities. However, *ad hoc* studies are needed to formally test this working model. Glycosylation of total IgG may capture information from non-HLA anti-donor antibodies that are hard to capture in routine clinical testing, or it may reflect a more systemic state of inflammation. Further studies are necessary to confirm and investigate these hypotheses.

In conclusion, we found a significant, independent correlation between total IgG glycosylation and AMR in kidney transplant recipients. More specifically, we showed the association of IgG bisecting GlcNAc regardless of the presence of DSA, with glomerulitis and chronic glomerulopathy in graft biopsy. Therefore, our findings provide the rationale for larger studies testing the hypothesis that total IgG glycosylation can be used as a biomarker for the risk of aAMR and caAMR. Strategies to target IgG glycosylation may also be leveraged therapeutically to improve the outcomes of transplant recipients.

## Data Availability

The original contributions presented in the study are included in the article/supplementary material, further inquiries can be directed to the corresponding authors.
