# Supplementary material for: Not all antibodies are created equal: total IgG glycosylation and severity of antibody-mediated rejection in kidney transplantation
Source: Transpl Int. 2026 Jun 12;39:16461. doi: 10.3389/ti.2026.16461 (PMC13315302; doi:10.3389/ti.2026.16461)
Supplement: Supplementary file 1 [file Supplementaryfile1.docx]

**SUPPLEMENTARY MATERIALS**

**Supplementary Figure 1: Key Structural Components and Modifications in N-Glycan Biosynthesis.** Illustration of the essential structural elements and modifications involved in the biosynthesis of N-glycans. Panel A highlights the dolichol phosphate (DolP) and its precursor role in the initial stages of glycan assembly and depicts high-mannose N-glycans attached to an asparagine (Asn) residue, representing an early intermediate in the pathway. Panel B shows the pentasaccharide core, a conserved structure in N-glycan synthesis. Panel C shows the branching process, marked by the addition of N-acetylglucosamine (GlcNAc) to the core. Panel D showcases the diversity of terminal sugar modifications, including glucose, mannose, sialic acid, GlcNAc, galactose, and fucose, which contribute to the functional variability of mature N-glycans.


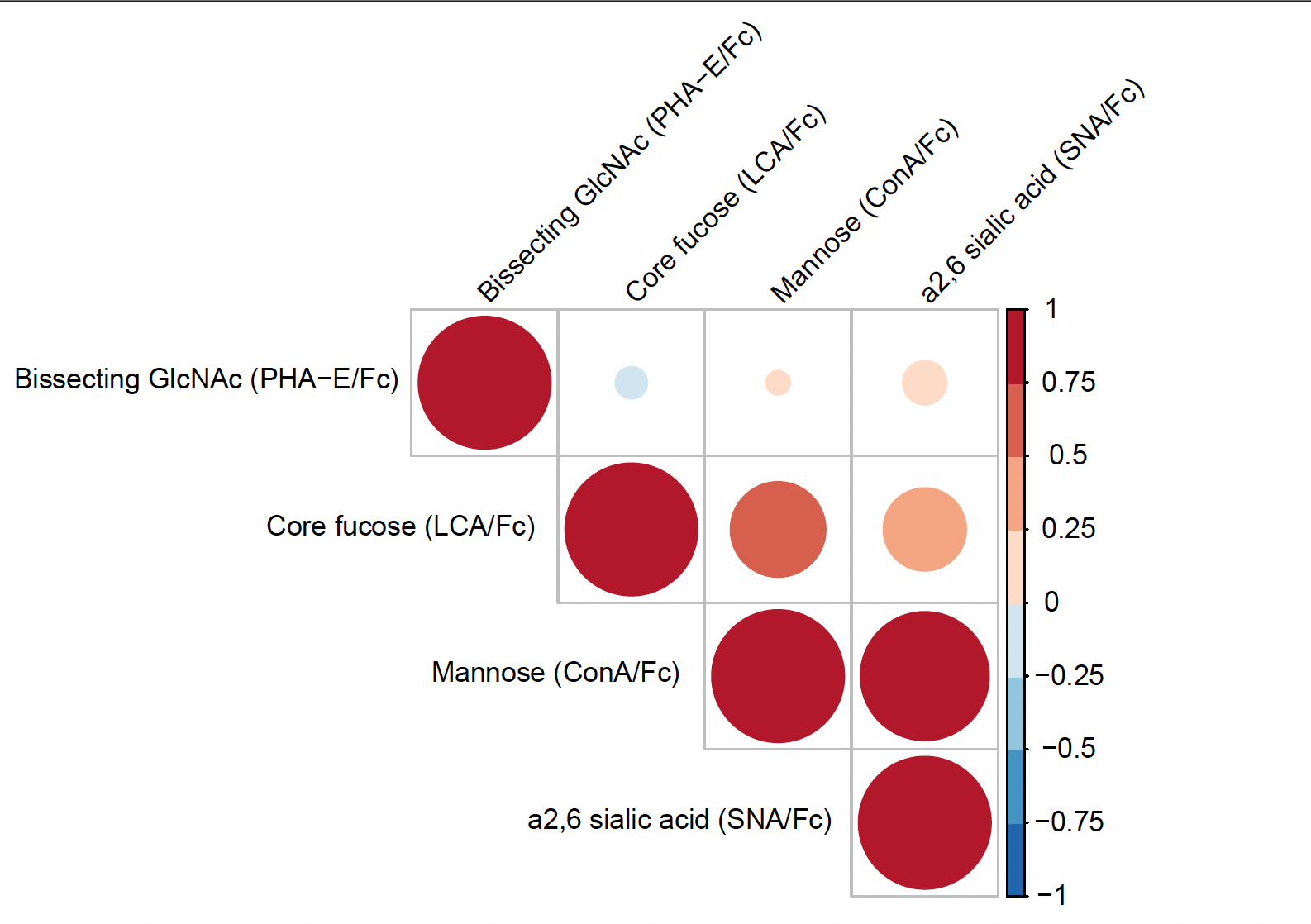


**Supplementary Figure 2: Comparative correlation of Lectin-Binding Profiles Normalized to Fc Levels.** The color of each dot reflects the strength and direction of the correlation (from −1 to 1), with warmer tones indicating stronger positive correlations and cooler tones indicating negative correlations. The size of the dots is proportional to the absolute value of the correlation coefficient, with larger dots representing stronger correlations regardless of direction.

ConA: Concanavalin A; LCA: Lens culinaris agglutinin; SNA: Sambucus nigra agglutinin; PHA-E: Phaseolus vulgaris Erythroagglutinin.

**
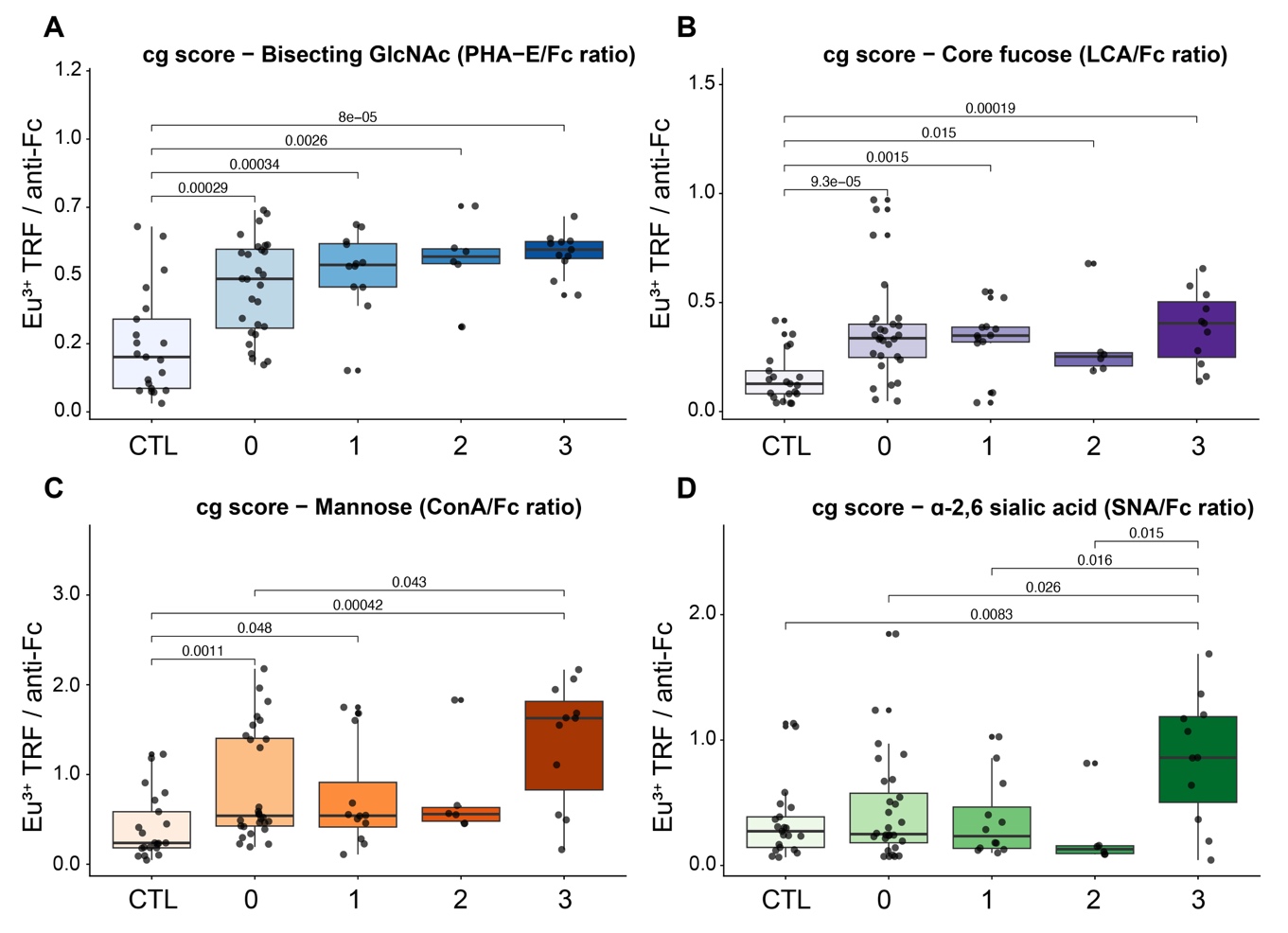
**

**Supplementary Figure 3: Associations between IgG post-translational modifications and the chronic lesion of rejection.** Box plots comparing the levels of bisecting GlcNAc (PHA-E/Fc ratio in panel A), core fucose (LCA/Fc ratio in panel B), mannose (ConA/Fc ratio in panel C), and α2,6-sialylation (SNA/Fc in panel D) with the severity score of chronic glomerulopathy (cg-score). p-values < 0.05 is indicated for each comparison.

ConA: Concanavalin A; cg: chronic glomerulopathy; c4d: complement factor 4d; g: glomerulitis; Eu³⁺ TRF / anti-Fc: europium-conjugated time-resolved fluorescence normalized to IgG; LCA: Lens culinaris agglutinin; PHA-E: Phaseolus vulgaris Erythroagglutinin; ptc: peritubular capilaritis; SNA: Sambucus nigra agglutinin.


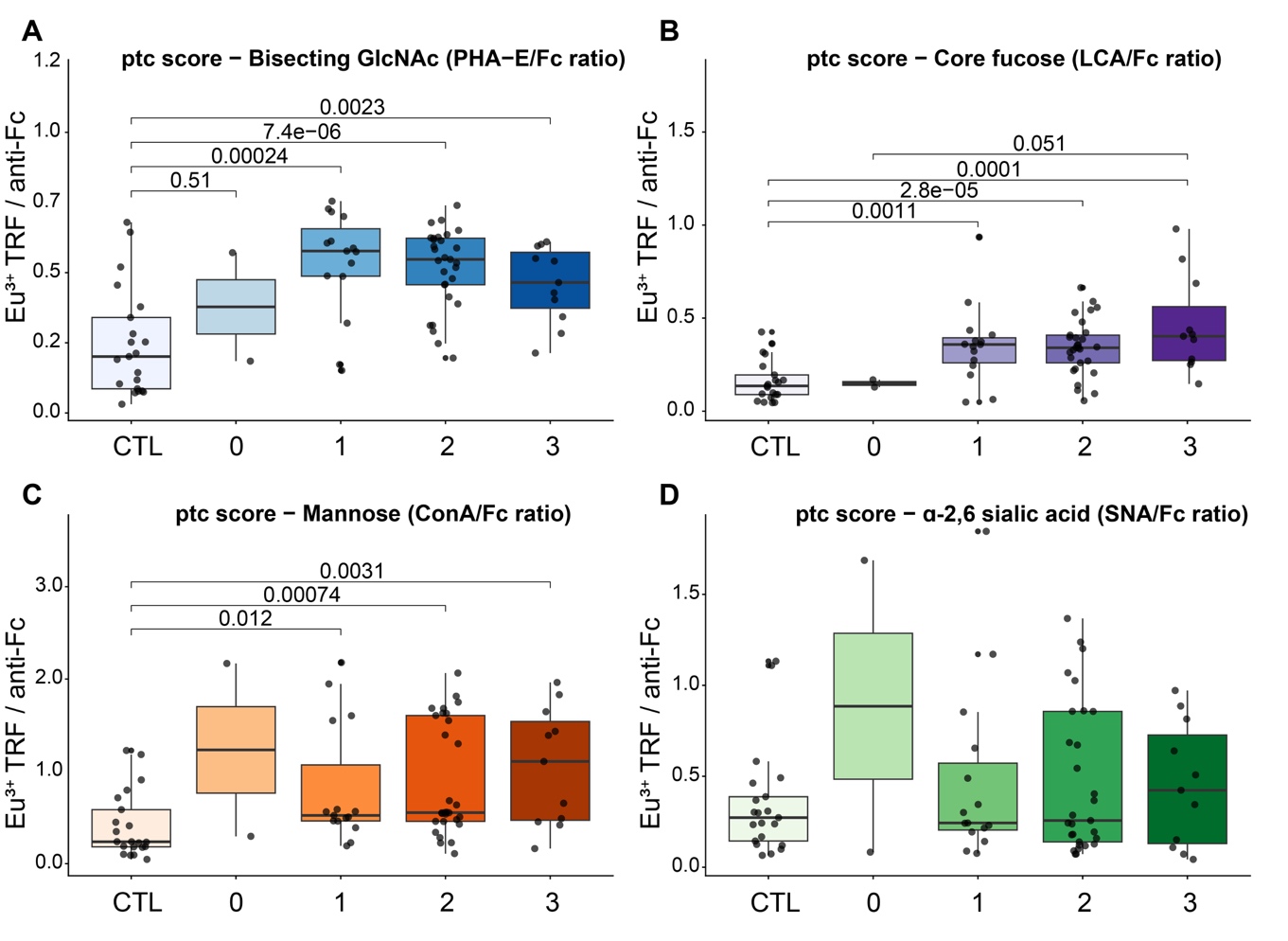


**Supplementary Figure 4: Associations between IgG post-translational modifications and the severity of peritubular capilaritis.** Box plots comparing the levels of bisecting GlcNAc (PHA-E/Fc ratio in panel A), core fucose (LCA/Fc ratio in panel B), mannose (ConA/Fc ratio in panel C), and α2,6-sialylation (SNA/Fc in panel D) with the severity score of peritubular capilaritis (ptc-score). p-values < 0.05 is indicated for each comparison.

ConA: Concanavalin A; cg: chronic glomerulopathy; c4d: complement factor 4d; g: glomerulitis; Eu³⁺ TRF / anti-Fc: europium-conjugated time-resolved fluorescence normalized to IgG; LCA: Lens culinaris agglutinin; PHA-E: Phaseolus vulgaris Erythroagglutinin; ptc: peritubular capilaritis; SNA: Sambucus nigra agglutinin.


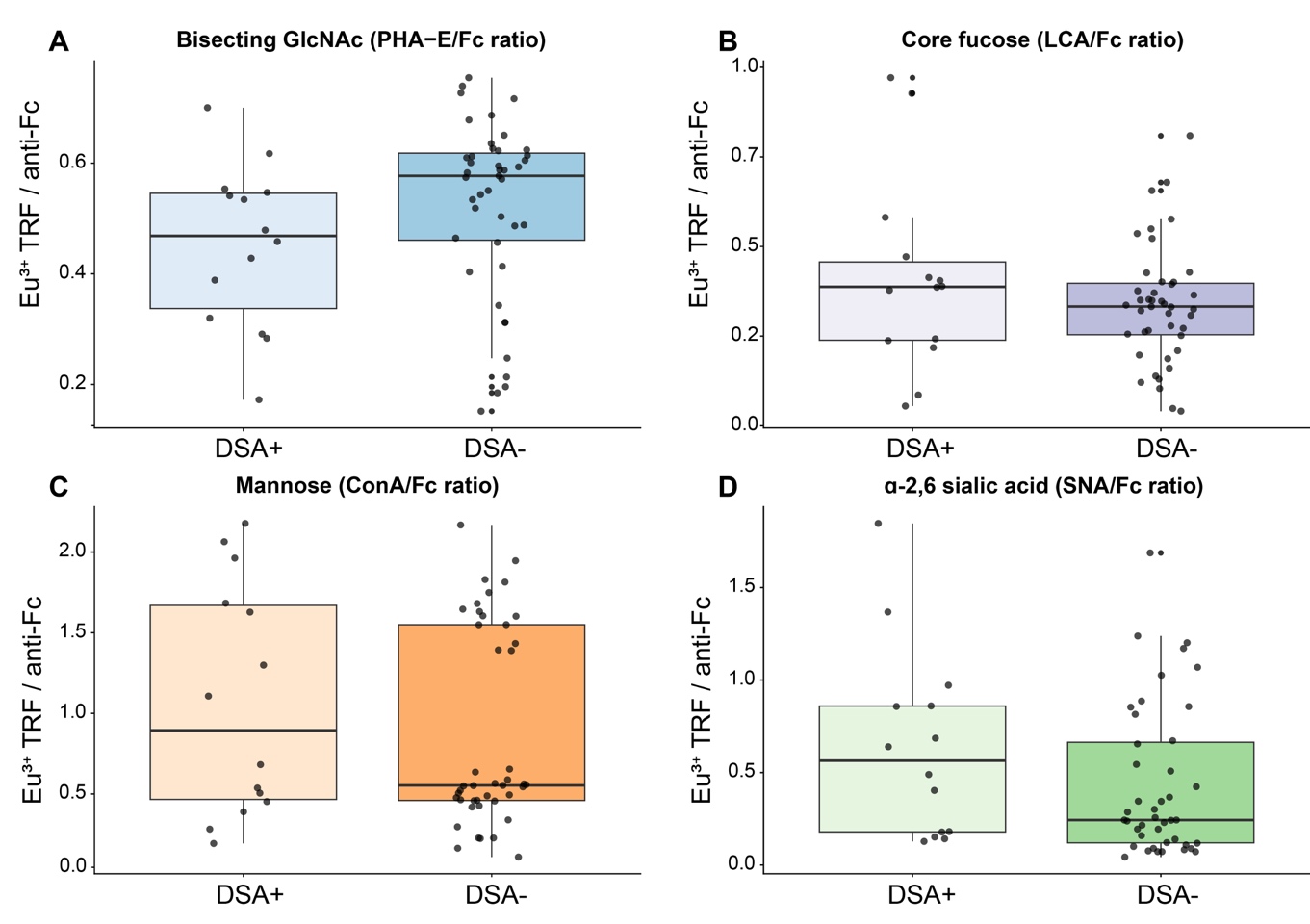


**Supplementary Figure 5: Associations between IgG post-translational modifications and the presence of Donor-specific Antibodies (DSA).** Box plots comparing the levels of bisecting GlcNAc (PHA-E/Fc ratio in panel A), core fucose (LCA/Fc ratio in panel B), mannose (ConA/Fc ratio in panel C), and α2,6-sialylation (SNA/Fc in panel D) with the severity score of peritubular capilaritis (ptc-score). p-values < 0.05 is indicated for each comparison.

ConA: Concanavalin A; cg: chronic glomerulopathy; c4d: complement factor 4d; g: glomerulitis; Eu³⁺ TRF / anti-Fc: europium-conjugated time-resolved fluorescence normalized to IgG; LCA: Lens culinaris agglutinin; PHA-E: Phaseolus vulgaris Erythroagglutinin; ptc: peritubular capilaritis; SNA: Sambucus nigra agglutinin.
